# Supplementary material for: Characterisation of GFAP-Expressing Glial Cells in the Dorsal Root Ganglion after Spared Nerve Injury
Source: Int J Mol Sci. 2023 Oct 25;24(21):15559. doi: 10.3390/ijms242115559 (PMC10647921; doi:10.3390/ijms242115559)
Supplement: Supplementary file 1 [file ijms-24-15559-s001.zip › ijms-2648839-supplementary.pdf]

Supplementary Materials

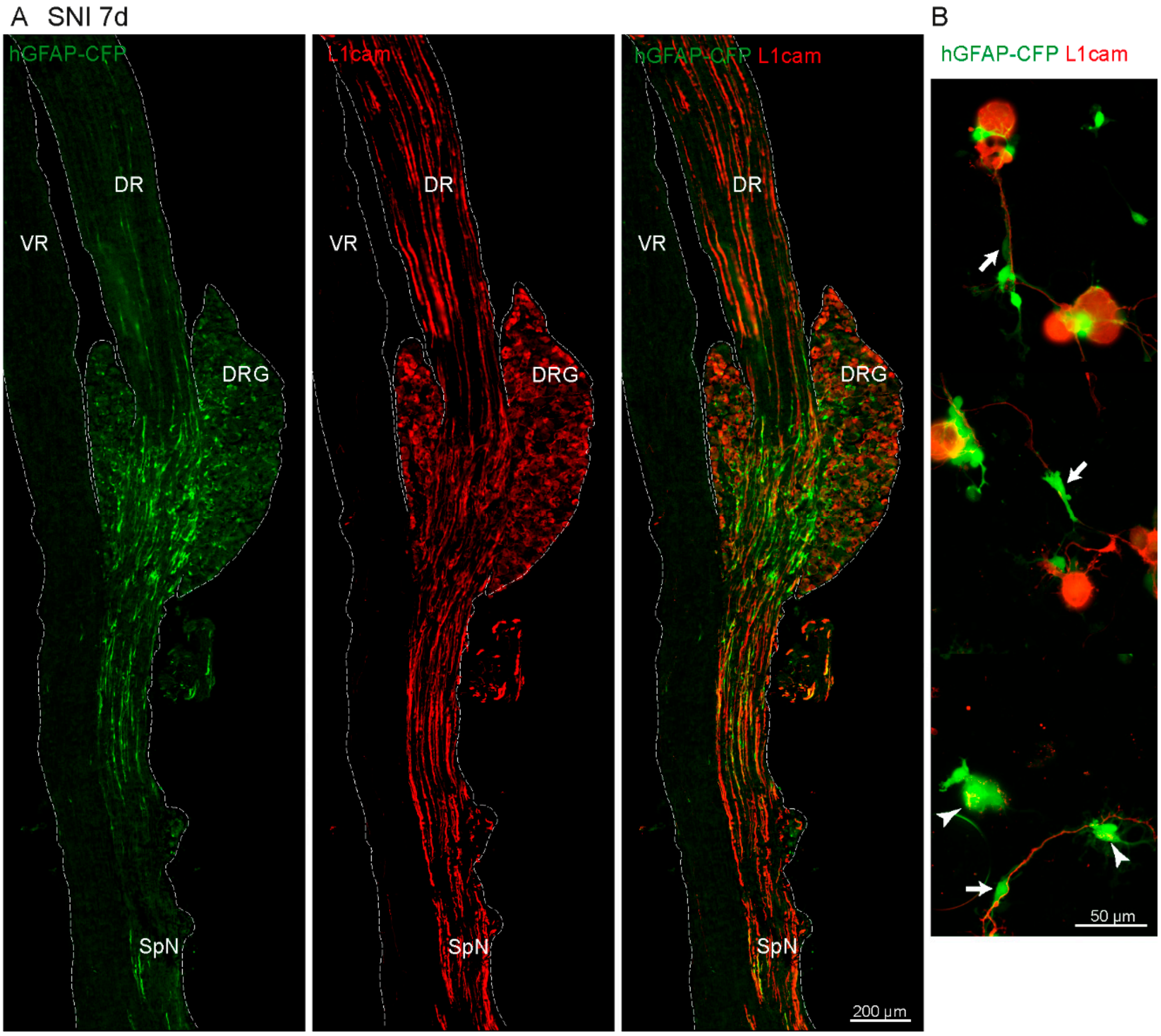

Supplementary figure S1: L1CAM expression in hGFAP-CF mice at 7 days after SNI. (A) Representative images of immunofluorescence staining against L1CAM in L4 DRG with ventral root (VR), dorsal root (DR) and spinal nerve (SpN) of the endogenous CFP signal (green), antibody staining (red) and merge. (B) Representative images of immunofluorescence staining against L1CAM (red) of dissociated culture of ipsilateral L3, L4 L5 DRG at 7 days after SNI, with arrowheads pointing to co-localisation and arrows to CFP+ cells (green) without co-localisation.

Supplemental table S1: List of reagents, solutions, and RRID references used in the current study.

Reagent or resource	Source (Company, City, State, Country)	Catalogue Number	RRID (Resource Identification Portal)
**Mice**			
Mouse line GFAP-CFP	Frank Kirchhoff, Max Planck Institute of Experimental Medicine, Göttingen, Germany	TgN(hGFAP-ECFP)-GCED	
**Materials / Equipment**			
PVDF membrane	BioRad, Hercules, CA, USA	#162-0177	
LAS4000 Fuji system (ImageQuant LAS 4000)	GE Healthcare, Chicago, IL, USA	LAS4000	
Zeiss Axio Scan.Z1 slide scanner	Zeiss, Oberkochen, Germany		
Falcon 5 mL Round Bottom Polystyrene Test Tube, with Cell Strainer Snap Cap	Corning, Corning, NY, USA	352235	
MoFlo Astrios EQ	Beckman Coulter, Brea, CA, USA	#B25982	
Thermanox plastic coverslips	Nunc, NY, Rochester	#174950	
Multiclamp Axon Amplifier 700B	Molecular Devices, San Jose, CA, USA	#700B	
Digidata 1440A	Molecular Devices, San Jose, CA, USA	#1440A	
CoolLED pE-340 fura	CoolLED Ltd, Andover, UK	#pE-340 fura	
LED eGFP pE-300 filterset	CoolLED Ltd, Andover, UK	#E3990113, Exciter: 460/30; Emitter: 520/40	
ORCAFlash2.8, model C11440-10C	Hamamatsu Photonics, Shizuoka, Japan	#820504	
Olympus BX63 fluorescent microscope	Olympus, Tokyo, Japan	BX63	
TH4 halogen lamp power supply unit	Olympus, Tokyo, Japan	TH4	
MF200-2 microforge	World Precision Instruments, Sarasota, FL, USA	MF200-2	
H4 platinum/iridium wire	World Precision Instruments, Sarasota, FL, USA	MF-200 H4	
Borosilicate patch pipettes with filament	Sutter Instruments, Novato, CA, USA	#BF150-86-7.5HP	
W30S-LED Revelation III	LW Scientific, Lawrenceville, GA, USA	W30S	
Perfusion valve controller VC-6	Warner Instrument, Hamden, CT, USA	VC-6	
Gilson minipuls suction system	Gilson, Middleton, WI, USA		
Nalgene 4 mm syringe filters	Thermo Fisher Scientific Inc., Waltham, MA, USA	176-0020	
SevenCompact S210	Mettler Toledo, Columbus, OH, USA	S210	
Osmometer 3320	Advanced Instruments Inc, Norwood, MA, USA	3320	
**Software**			
Fiji (ImageJ Software)	Schindelin et al., 2012		SCR_002285
Zeiss 3.1 Blue software	Zeiss, Oberkochen, Germany		SCR_013672

CellSens v3.2	Olympus, Tokyo, Japan		SCR_014551
pClamp 10 Software	Molecular Devices, San Jose, CA, USA		SCR_011323
GraphPad Prism 10	GraphPad Software, Boston, MA, USA		SCR_002798
**Antibodies**			
Rabbit anti-GFAP	Aligent, Santa Clara, CA, USA	Z0334	AB_10013382
Rabbit anti-Kv1.1	Thermo Fisher Scientific Inc., Waltham, MA, USA	PA5-102673	AB_2852070
Rabbit anti-Kv1.6	Thermo Fisher Scientific Inc., Waltham, MA, USA	PA5-19498	AB_10979590
Rabbit anti-α-tubulin	Sigma-Aldrich Inc., Saint-Louis, MO, USA	#T5168	AB_477579
Rabbit anti-iba1	Wako Pure Chemical Industries Ltd, Richmond, VA, USA	19741	AB_839504
Rabbit anti-Ki67	Merck Millipore, Burlington, MA, USA	ab9260	AB_2142366
Rabbit anti-GS	Abcam, Waltham, MA, USA	ab64613	AB_1140869
Rabbit anti-Fabp7	Thermo Fisher Scientific Inc., Waltham, MA, USA	PA5-24949	AB_2542449
Rabbit anti-connexin43	Cell Signaling Techniology, Danvers, MA, USA	3512	AB_2294590
Guinea pig anti-Kir4.1	Thermo Fisher Scientific Inc., Waltham, MA, USA	PA5-111798	AB_2857207
Rabbit anti-L1cam	Merck Millipore, Burlington, MA, USA	MAB5272	AB_2133200
Rabbit anti-ATF3	Abcam, Waltham, MA, USA	ab207434	AB_2734728
Chicken anti-NeuN	Neuromics, Edina, MN, USA	CH23022	AB_2737147
HRP-linked goat anti-rabbit	Aligent, Santa Clara, CA	P0448	AB_2617138
HRP-linked goat anti-mouse	Aligent, Santa Clara, CA	P0447	AB_2617137
Donkey Cy3 anti-rabbit	Jackson ImmunoResearch, Ely, UK	711-165-152	AB_2307443
Goat Alexa Fluor 594 anti-guinea pig	Molecular Probes, Eugene, OR, USA	A11076	AB_141930
Goat Alexa Fluor 594 anti-chicken	Molecular Probes, Eugene, OR, USA	A11042	AB_2534099
Donkey Cy3 anti-goat	Jackson ImmunoResearch, Ely, UK	705-165-147	AB_2307351
Goat Alexa Fluor 350 anti-rabbit	Molecular Probes, Eugene, OR, USA	A21068	AB_141378
**Reagents**			
Isoflurane	Piramal, Mumbai, Maharashtra, India	G45C19A	
Pentobarbital	Streuli Pharma, Uznach, Switzerland	V102013	
Hanks' Balanced Salt solution	Sigma-Aldrich Inc., Saint-Louis, MO, USA	H6648-500ML	
Tris	AppliChem, Darmstadt, Germany	#A1086	
Complete protease inhibitor cocktail	Roche, Basel, Switzerland	#11697498001	
Triton X-100	Sigma-Aldrich Inc., Saint-Louis, MO, USA	#T9284	
SDS (Sodium Dodecyl Sulfate)	Sigma-Aldrich Inc., Saint-Louis, MO, USA	#L4390	
Bovine Serum Albumin	Sigma-Aldrich Inc., Saint-Louis, MO, USA	A3294	
Protein Assay Dye Reagent Concentrate	Bio-Rad, Hercules, CA, USA	500-0006	
SuperSignal West Dura Extended Duration Substrate	Thermo Fisher Scientific Inc., Waltham, MA, USA	#34075	

Paraformaldehyde	Sigma-Aldrich Inc., Saint-Louis, MO, USA	P6148	
Phosphate Buffered Saline (PBS)	Bischel AG, Bern, Switzerland	100 0 325	
Sucrose (D+ Saccharose)	AppliChem, Darmstadt, Germany	#A2211	
Tissue-Tek O.C.T. Compound	Sakura Finetek, Alphen aan den Rijn, Netherlands	#4583	
Normal goat serum	Vector Laboratories, Newark, CA, USA	S-1000	
Normal horse serum	Vector Laboratories, Newark, CA, USA	S-2000	
Mowiol 4–88 medium	Calbiochem, San Diego, CA, USA	#475904	
Dispase II	Roche, Basel, Switzerland	4942078001	
Collagenase A	Roche, Basel, Switzerland	10103578001	
DMEM	Gibco, Billings, MT, USA	41965-039	
Fetal Bovine Serum	Gibco, Billings, MT, USA	10082	
Penicillin/Streptomycin	Sigma-Aldrich Inc., Saint-Louis, MO, USA	P0781	
Poly-D-Lysine	Sigma-Aldrich Inc., Saint-Louis, MO, USA	P0899	
4-Aminopyridine (4AP)	Sigma-Aldrich Inc., Saint-Louis, MO, USA	275875-1G	
NaCl (Sodium Chloride)	Sigma-Aldrich Inc., Saint-Louis, MO, USA	#S9625	
CaCl2 (Calcium chloride dihydrate)	Merck Millipore, Burlington, MA, USA	#102382	
HEPES	AppliChem, Darmstadt, Germany	#A1069	
Glucose D-(+)	Sigma-Aldrich Inc., Saint-Louis, MO, USA	#G7021	
EGTA (Ethylene Glycol Tetra Acetic Acid)	Sigma-Aldrich Inc., Saint-Louis, MO, USA	#E4378	
KCl (Potassium Chloride)	Sigma-Aldrich Inc., Saint-Louis, MO, USA	#P9333	
NaOH (Sodium hydroxide)	Sigma-Aldrich Inc., Saint-Louis, MO, USA	#S8045	
KOH	Sigma-Aldrich Inc., Saint-Louis, MO, USA	P5958-250G	
MgCl2-6H2O (Magnesium chloride hexahydrate)	Merck Millipore, Burlington, MA, USA	#105832	

Supplemental table S2: List of statistical test for significance for each figure.

Fig.	Statistical test	N	Stat-value	P-value (Two-tailed)
**Figure 1**				
1C	Mean, S.D., S.E.M.	N=6 DRG cultures; 24 images	78.43, 12.07, 4.929	-
**Figure 2**				
2D	2way ANOVA with posthoc Dunnett's multiple comparisons against naïve group	L3 N=4 mice; 108 images L4 N=4 mice; 96 images L5 N=3 mice; 86 images	DRG F (2, 45) = 13.56<br>SNI time course F (4, 45) = 21.73<br>Interaction F (8, 45) = 3.012	DRG p<0.0001<br>SNI time course p<0.0001<br>Interaction p=0.0085
2E	2way ANOVA with posthoc Dunnett's multiple comparisons against naïve group	L3 N=4 mice; 57 images L4 N=4 mice; 53 images L5 N=3 mice; 41 images	DRG F (2, 40) = 27.93<br>SNI time course F (4, 40) = 21.91<br>Interaction F (8, 40) = 7.193	DRG p<0.0001<br>SNI time course p<0.0001<br>Interaction p<0.0001
2F	2way ANOVA with posthoc Dunnett's multiple comparisons against naïve group	L3 N=4 mice; 57 images L4 N=4 mice; 53 images L5 N=2 mice; 41 images	DRG F (2, 35) = 0.4356<br>SNI time course F (4, 35) = 4.099<br>Interaction F (8, 35) = 2.264	DRG p=0.6503<br>SNI time course p=0.0079<br>Interaction p=0.0457
2G	Unpaired student's T-test	N=5 mice	t=2.832, df=8	p=0.0221
**Figure 3**				
3D	2way ANOVA with posthoc Dunnett's multiple comparisons against naïve group	L3 N=3-4 mice; 51 images L4 N=3-4 mice; 43 images L5 N= 4mice;	DRG F (2, 43) = 48.42<br>SNI time course F (4, 43) = 37.52<br>Interaction F (8, 43) = 8.411	DRG p<0.0001<br>SNI time course p<0.0001<br>Interaction p<0.0001

		37 images		
3E	2way ANOVA	N=8 DRG (L3, 51 images and L4, 43 images from 4 mice)	Neuron type F (1, 42) = 19.17<br>SNI time course F (2, 42) = 1.185<br>Interaction F (2, 42) = 0.02232	Neuron type p<0.0001<br>SNI time course p=0.3157<br>Interaction p=0.9779
**Figure 4**				
4G	One-way ANOVA with posthoc Dunnett's multiple comparisons against naïve group	N=4 mice; 111 images	F (4, 15) = 6.671	p=0.0027
4H	One-way ANOVA with posthoc Dunnett's multiple comparisons against naïve group	N=4 mice; 111 images	F (4, 15) = 7.825	p=0.0013
4I	One-way ANOVA	N=4 mice; 111 images	F (4, 15) = 1.068	p=0.4066
**Figure 5**				
5C	Paired student's T-test	N=4 mice; 16 images	t=6.551, df=3	p=0.0072
**Figure 6**				
6B	2way ANOVA	Contra N=7 cells<br>Ipsi N=24 cells<br>Ipsi + 4AP N=8 cells	Group F (2, 722) = 48.37<br>Voltage F (18, 722) = 43.04<br>Interaction F (36, 722) = 1.189	Group p<0.0001<br>Voltage p<0.0001<br>Interaction p=0.2102
6C	2way ANOVA	Contra N=25 cells<br>Ipsi N=35 cells	Group F (1, 1450) = 8.568<br>Voltage F (24, 1450) = 90.60<br>Interaction F (24, 1450) = 0.2565	Group p=0.0035<br>Voltage p<0.0001<br>Interaction p>0.9999
6D	Unpaired student's T-test	Contra N=23 cells<br>Ipsi N=35 cells	t=1.766, df=56	p=0.0829
6H	Paired student's T-test	N=5 samples of CFP+ cells	t=0.7314, df=4	p=0.5051
6I	Paired student's T-test	N=5 samples of CFP+ cells	t=0.8691, df=4	p=0.4338
